# Tris(2-Pyridylmethylamine)V(O)_2_ Complexes as Counter Ions of Diprotonated Decavanadate Anion: Potential Antineoplastic Activity

**DOI:** 10.3389/fchem.2022.830511

**Published:** 2022-02-16

**Authors:** Nidia D. Corona-Motolinia, Beatriz Martínez-Valencia, Lisset Noriega, Brenda L. Sánchez-Gaytán, Francisco J. Melendez, Amalia García-García, Duane Choquesillo-Lazarte, Antonio Rodríguez-Diéguez, María Eugenia Castro, Enrique González-Vergara

**Affiliations:** ^1^ Centro de Química del Instituto de Ciencias, Benemérita Universidad Autónoma de Puebla, Puebla, Mexico; ^2^ Facultad de Ciencias Químicas, Benemérita Universidad Autónoma de Puebla, Puebla, Mexico; ^3^ Departamento de Química Inorgánica, Facultad de Ciencias, Universidad de Granada, Granada, Spain; ^4^ Laboratorio de Estudios Cristalográficos, IACT, CSIC-UGR, Granada, Spain

**Keywords:** decavanadate, vanadium (V) dioxido compounds, TPMA, DFT calculations, molecular docking, antineoplastic activity

## Abstract

The synthesis and theoretical-experimental characterization of a novel diprotanated decavanadate is presented here due to our search for novel anticancer metallodrugs. Tris(2-pyridylmethyl)amine (TPMA), which is also known to have anticancer activity in osteosarcoma cell lines, was introduced as a possible cationic species that could act as a counterpart for the decavanadate anion. However, the isolated compound contains the previously reported vanadium (V) dioxido-tpma moieties, and the decavanadate anion appears to be diprotonated. The structural characterization of the compound was performed by infrared spectroscopy and single-crystal X-ray diffraction. In addition, DFT calculations were used to analyze the reactive sites involved in the donor-acceptor interactions from the molecular electrostatic potential maps. The level of theory mPW1PW91/6–31G(d)-LANL2DZ and ECP = LANL2DZ for the V atom was used. These insights about the compounds’ main interactions were supported by analyzing the noncovalent interactions utilizing the AIM and Hirshfeld surfaces approach. Molecular docking studies with small RNA fragments were used to assess the hypothesis that decavanadate’s anticancer activity could be attributed to its interaction with lncRNA molecules. Thus, a combination of three potentially beneficial components could be evaluated in various cancer cell lines.

## Introduction

Polyoxometalates (POM) is a special class of discrete, anionic metal-oxygen clusters considered soluble oxide fragments (Bijelic et al., 2018). Numerous organic and inorganic species can be incorporated into POM frameworks, resulting in a wide range of shapes, sizes, and nuclearities, as well as a wide range of catalytic, material science, photochemical, magnetic, and biological properties that have been shown to have excellent antibacterial, antiviral, and antitumoral activity (Gumerova and Rompel, 2020). Decavanadate, as one of the most promising POM members, has received much attention in recent decades due to their pharmacological and biochemical properties, as they play a critical role in biological systems because of their capacity to interact with proteins, enzymes, and cell membranes (Aureliano et al., 2021; Aureliano et al., 2022). More than forty years ago, vanadate was discovered as an impurity in commercial ATP derived from horse skeletal muscle by inhibiting sodium pump-action (Cantley et al., 1977). Decavanadate’s high negative charge allows it to interact with a wide range of molecules, including proteins, counterions, and lipid structures, influencing a variety of biological processes such as muscle contraction, calcium homeostasis, necrosis, actin polymerization, oxidative stress markers, and glucose uptake (Aureliano, 2009). As a result, numerous compounds based on decavanadate and cationic organic ligands have been published in recent years, which have been shown to lower blood sugar levels (Garcia-Vicente et al., 2007; Treviño et al., 2015; Treviño et al., 2018; Treviño and González-Vergara, 2019), induce neuronal and cognitive restoration mechanisms while treating metabolic syndrome (Diaz et al., 2021), and inhibit the growth of protozoan parasites (Li et al., 2010; Silva-Nolasco et al., 2020). The cytotoxic or differentiating activity of oxidovanadium complexes with organic ligands against various cancer cell types is well known (Kioseoglou et al., 2015; Crans et al., 2018; Crans et al., 2019; Redher, 2020; Samart et al., 2020; Macedo Alves de Lima et al., 2021; Pessoa et al., 2021). However, their limited use in clinical trials is due to concerns about long-term toxicity caused by a lack of data, as well as complex speciation (ligand exchange and redox processes). Nevertheless, a recent 300 mg/kg dose of [VO(HSHED)dtb] complex administration to mice showed no signs of toxicity. The complex was less toxic than orthovanadate salt, indicating that the compound was not broken down during administration (Lima et al., 2021). The low toxicity is due to the redox properties obtained by coupling the redox-active ligand 3,5-di (tert-butyl)catechol with the hydrolytic stability of the [VO(HSHED)dtb], which prevents vanadate and catechol ligand formation. These findings imply that protecting cationic vanadium species may help to increase activity while decreasing toxicity. Although anionic vanadium compounds can survive the bloodstream and enter cells through various mechanisms, it is possible that they primarily generate V1, V2, and V4 vanadates through speciation (Crans et al., 2018; Redher, 2020). As a result, studying the cyclo-tetravanadate anion is critical because it will be the dominant species at physiological pH (Sanchez-Lara et al., 2018). Anticancer properties of vanadium(V) anionic compounds have been discovered (Cheng et al., 2018; Silva-Nolasco et al., 2020). Mg(H_2_O)_6_(C_4_N_2_H7)_4_V_10_O_28_•4H_2_O and (C_7_H_10_N)_4_ [H_2_V_10_O_28_]•2H_2_O were recently shown to have dose-dependent antiproliferative activity against human cancer cells U87, IGR39, and MDA-MB-231 (Ksiksi et al., 2021; Louati et al., 2021). The *in vitro* encapsulation of V(IV)-curcumin-bipyridine (VCur) with magnetic cationic liposomes improves the compound’s stability and solubility in physiological media (Halevas et al., 2019). Biocompatible vanadium nitride nanosheets absorb a significant amount of near-infrared (NIR) light, which can be used to image cancer tissue and generate reactive oxygen species upon NIR excitation, effectively killing cancer tissue. Recent research on eleven vanadium species, compounds, and materials in human melanoma cell lines has revealed intriguing antitumor properties in response to a variety of effects, including: 1) cell viability; 2) cell morphology changes and apoptosis; 3) cell-cycle arrest; 4) ROS production; 5) mitochondrial dysfunction; 6) protein expression; and 7) *in vivo* tumor regression and survival rates. As a result, it was determined that critical questions would be addressed this decade to advance the use of vanadium compounds in cancer treatment (Amante et al., 2021). According to recent research, these compounds may effectively treat solid tumors, DNA and RNA viruses, and drug-resistant bacteria. Antibacterial modes of action for various POVs include changes in cell structure, interference with the ion transport system, inhibition of mRNA synthesis, disruption of metabolic pathways, and communication mechanisms. POVs have also been shown *in vitro* to reduce mitochondrial respiration. Researchers should investigate vanadium-containing POV-based nanohybrids rather than pure POVs (Aureliano et al., 2021).

On the other hand, the first metal complex with Tris(2-pyridylmethyl)amine (TPMA) was discovered in 1969 with Nickel (II) (da Mota et al., 1969). Many complexes have been reported since then. (TPMA) is a neutral tripodal nitrogen-based ligand frequently complex with many transition metals. The Cambridge Crystallographic Database contains over 500 structures of metals ranging from groups 1 to 13, with many examples from the lanthanide and actinide families (Eckenhoff and Pinthauer 2010). TPMA is a chelator that usually forms a tetradentate bond with the metal; however, in some cases, and tridentate coordination via pyridyl nitrogen arm dissociation has been observed. TPMA has attracted much attention as a preferred ligand in copper complexes that resemble specific metalloenzymes involved in oxygen activation (Karlin et al., 1982). Furthermore, a series of oxoperoxovanadium(V) complexes tris(2-pyridylmethyl)amine) were identified as functional models for vanadium haloperoxidase enzymes (Colpas et al., 1996). Copper complexes are also very active in atom transfer radical addition (ATRA) (Pintauer, 2015) and polymerization (ATRP) (Matyjaszewski and Xia, 2001). An integrated approach was recently used to investigate the biological reactivity of a novel artificial nuclease [Cu(TPMA) (Phen)](ClO_4_)_2_ within liposome and cellular models using free and encapsulated drug forms. A nanoscale hollow pH-sensitive drug-delivery system was successfully used and released (Toniolo et al., 2018). TPMA ligands have recently gained popularity in developing novel architectures and functional systems. The ability to form stable metal complexes with a wide range of metals and the benefits conferred by the ligand’s helical orientation around the metal drives this growing interest (Bravin et al., 2021). In 2005, Tajika et al. reported the first mononuclear oxovanadium (IV) and dioxovanadium(V) complexes of TPMA (Tajika et al., 2005). The crystal structures of three oxovanadium (IV) complexes [VO(SO_4_) (tpma)] [VOCl(tpma)]PF_6_, or [VOBr(tpma)]PF_6_, and a dioxidovanadium(V) complex [V(O)_2_ (tpma)], were determined. The tertiary nitrogen of the TPMA ligand always occupies the trans-to-oxo site, according to PF_6_. The same group of researchers looked into the oxidation reactions of -terpinene and 2,6-di-tert-butylphenol by vanadium (V). For the oxidation reactions of -terpinene and 2,6-di-tert-butylphenol, the dioxidovanadium(V) complex was more reactive than the corresponding oxo-peroxo vanadium(V) complex. The insulin-mimetic potential of bis- and tris(pyridyl)amine-oxidovanadium complexes has been investigated (Nilsson et al., 2009). TPMA has also been proposed as a next-generation chelator for studying intracellular mobile zinc. In cuvettes, live cells, and brain tissue, TPMA sequesters mobile zinc with faster kinetics. TPMA also reduces *in vitro* cytotoxicity during time windows that are frequently used in studies of mobile zinc biology. TPMA improves its zinc-binding kinetics and reduces the cytotoxicity of chelator treatment. The potential of TPMA as an alternative chelator for mobile zinc in biological samples emphasizes the importance of further investigating its applications in zinc biology and other metal studies (Huang et al., 2013).

Recently, our research group has been involved in the synthesis and experimental and theoretical characterization of decavanadates and cyclo-tetravanadates as potential metallo-prodrugs for the treatment of diabetes and cancer (Sánchez-Lara et al., 2015; Sánchez-Lara et al., 2019; Sánchez-Lara et al., 2021), (Martínez-Valencia et al., 2020), (Martínez-Valencia et al., 2020), (Corona-Motolinia et al., 2020), and (García-García et al., 2021). We attempted to synthesize a protonated tris(2-pyridoniummehtyl)amine decavanadate to find new candidates under mild hydrothermal conditions. Surprisingly, the compound crystallized as a diprotonated decavanadate with dioxidovanadium(V) coordinated to the TPMA molecule. As a result, the synthesis, experimental, and theoretical characterization of the complex [VO_2_(tpma)]_4_ [H_2_V_10_O_28_] are described in this paper. The hypothesis that decavanadate’s anticancer activity could be attributed to its interaction with lncRNA molecules was tested using molecular docking studies with small RNA molecules. As a result, a combination of three potentially beneficial components could be tested in different cancer cell lines, regardless of whether they break before or after crossing the cell membrane.

## Experimental Section

### Synthesis of [VO_2_(tpma)]_4_[H_2_V_10_O_28_] (1)

The compound was made starting from an aqueous solution of (0.500 g, 4.27 × 10^−3^ mol) of ammonium metavanadate in 30 ml of distilled water; it was solubilized with constant stirring and moderate heat. When completely homogeneous the pH was adjusted to 5.5 by adding acetic acid 1 M dropwise. A second solution containing tpma in (0.250 g, 0.86 × 10^−3^ mol) in 15 ml of distilled water was added to the orange vanadium solution. The resulting solution was filtered and left at room temperature; adjusting the pH to 5.35 caused the precipitation of a microcrystalline yellow compound. The yield of the first crop of crystals was 53%. Yellow (gold) crystals were obtained after several days.

### Computational Methods

Theoretical calculations based on the density functional theory (DFT) (Hohenberg and Kohn, 1964) were used to obtain the structural and electronic properties of the compound [VO_2_(tpma)]_4_ [H_2_V_10_O_28_]·(**1**). The optimized geometry of **Compound 1** was obtained using the functional mPW1PW91. This functional use the Perdew-Wang exchange as modified by Adamo and Barone combined with PW91 correlation (Adamo and Barone, 1998). The split-valence 6–31G(d) basis set (Rassolov et al., 1998), including a single set of Gaussian polarization functions, was used for the C, H, O, and N atoms. A LANL2DZ basis set (Hay and Wadt, 1985) and an effective core potential (ECP) was used for the V atom. The ECP replaces the effects of the inner core electrons with a pseudopotential specific for transitions metal atoms. The aqueous solvent effect was calculated with the universal solvation model based on solute electron density (SMD) (Manerich et al., 2009). In addition, the molecular electrostatic potential (MEP) was analyzed. All the calculations were carried out with the Gaussian16 program (Frisch et al., 2016), and the results were visualized with the GaussianView 6.0.16 program (Dennington et al., 2016). In addition, the main noncovalent interactions in **Compound 1** were characterized using the atoms in molecules (AIM) approach, with AIMAll software (Keith, 2019), and the Hirshfeld surface analysis, using CrystalExplorer 17.5 software (Turner et al., 2017).

### Molecular Docking Analysis

Molecular docking analysis was carried out with the semi-flexible methodology, where the RNAs fragments were considered a rigid entity, while flexibility was allowed for the ligands [VO_2_(tpma)]+, and diprotonated decavanadate. The preparation of the macromolecule and the ligand was performed through the Autodock Tools 1.5.6 software (Morris et al., 2009), which includes polar hydrogens and empirical particles of atomic charges (Gasteiger–Marsili method). Different grid box sizes were used for each RNA molecule that encloses the entire fragment: for 6PK9, 60, 106, and 60 Å were used; 70, 126, and 70 Å for 2JXV fragment; and 94, 76, and 66 Å were used for 2MNC. In addition to those fragments, one DNA structure was considered in the docking study, 151D, with sizes of 70, 70, and 120 Å. The grid spacing for all the docking calculations was set to the default 0.375 Å value, using the Lamarckian genetic algorithm (LGA) searching methods. The parameters for the vanadium atom were the sum of VDW radii of two similar atoms (3.14 Å), plus the VDW well depth (0.016 kcal/mol), plus the atomic solvation volume (12.0 Å^3^), plus the atomic solvation parameter (−0.00110). The H-bond radius of the heteroatom in contact with hydrogen (0.0 Å), the well depth of the H-bond (0.0 kcal/mol), and different integers indicate the type of H-bonding atom and indexes for the generation of the autogrid map (0, −1, −1, and 1, respectively). The program Discovery Studio by Biovia was used to visualize the docked structures.

## Results

### Crystal Structure of [VO_2_(tpma)]_4_[H_2_V_10_O_28_]·4H_2_O (1)

**FIGURE 1:**
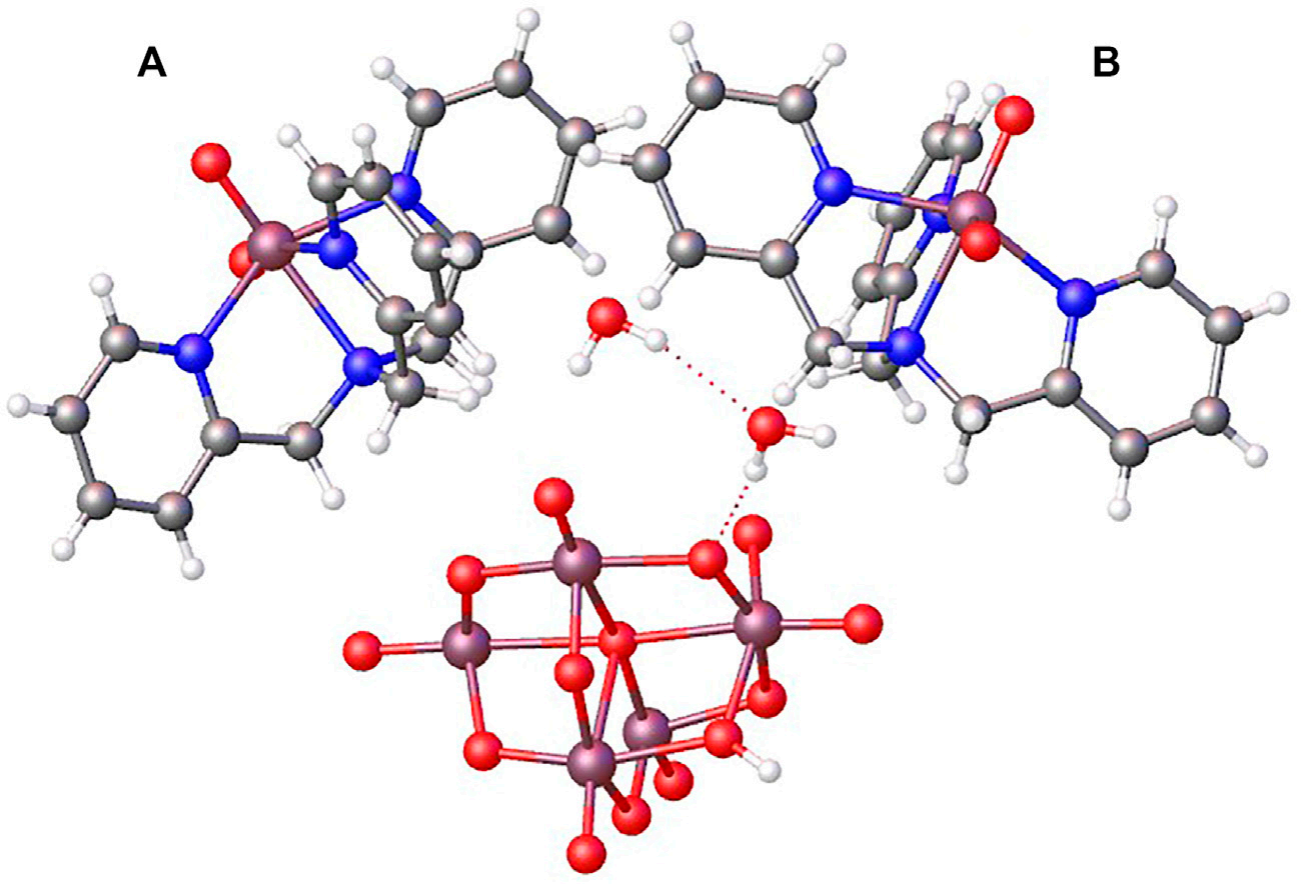
OLEX representation of the asymmetric unit containing half of the diprotonated decavanadate, two slightly different dioxidovanadium(V) tpma moieties **(A,B)**, and two crystallization water molecules.

The title compound is built up by mononuclear [VO_2_(tpma)]^+^ cations, diprotonated decavanadate anions [H_2_V_10_O_28_]^4-^, and crystallization water molecules. **Compound 1** crystallizes in the P2_1_/c space group of the monoclinic system and its asymmetric unit is composed of a half of a centrosymmetric decavanadate anion, two independent [VO_2_(tpma)]^+^ cations (named as A and B), and two water molecules of crystallization as seen in Figure 1. Table 1 contains the crystallographic data for Compound 1.

**TABLE 1:** Crystallographic data.

Compound	1
Empirical formula	C_72_H_82_N_16_O_40_V_14_
Formula weight (g·mol^−1^)	2,524.69
CCDC	2124254
Crystal system	Monoclinic
Space group	P2_1_/c
a (Å)	11.6736 (10)
b (Å)	23.7172 (17)
c (Å)	16.5914 (12)
α (°)	90
β (°)	95.791 (3)
λ (°)	90
Volume (Å^3^)	4,570.1 (6)
Z	2
Density (calcd) (g·cm^−3^)	1.835
μ(Mo/CuKα) (mm^−1^)	1.457
Temperature (K)	300
GoF on F^2^	1.049
R_1_ [1 > 2σ(I)]	0.0393
R_1_ [all data]	0.0731
wR_2_ [1 > 2σ(I)]	0.0781
wR_2_ [all data]	0.0917

The decavanadate unit is built up from five vanadium atoms generating, by an inversion center, a cluster of ten distorted edge-sharing VO_6_ octahedra containing sixty differentiated V-O bonds by their bond distances. V-Oc bonds, corresponding to bridging O atoms with coordination number 6, exhibit bond distances in the range of 2.1390(16)-2.3271(16) Å. Bond lengths of the three-coordinated oxygen atoms V-Ob1 range from 1.9162 (17) to 2.0670 (17) Å. The bridging two-coordinated oxygen atoms V-Ob2 bond lengths are within 1.6873 (17) and 2.0867 (18) Å. The eight terminal V=O_t_ bond lengths are the smallest ones with values in the range of 1.6033(19)-1.6111(18) Å. Lastly, the V-V distances are between 3.1053 (6) and 3.0171 (6) Å. The bond distances are similar to [V_10_O_28_]^6−^ units found in the literature (Kumagai et al., 2002; Huang et al., 2020). The excellent quality of X-ray diffraction data allowed to localize the hydrogen atoms within the decavanadate cluster in O10 atom, led by an inversion center to diprotonated decavanadate units [H_2_V_10_O_28_]^4−^. According to Brown and Altermatt, the standard bond valence summations confirmed this fact (Brown and Altermatt, 1985). The sums Σs = Σ(R/1.791)^−5.1^, where R is the V-O distance in Å and s is the bond valence, are in the range of 1.71–1.98 for all oxygen atoms except 1.22 for the O10 atom. This agrees with previously reported dehydrogenated decavanadate anions; since doubly and triply bonded oxygen atoms are the more basic sites, they are the first to protonate (Howarth and Jarrold, 1978; Capparelli et al., 1986; Correia et al., 2004).

On the other hand, the structure shows [VO_2_(tpma)]^+^ cations around decavanadate units. In the asymmetric unit appears two crystallographically independent [VO_2_(tpma)]^+^ cations (named A and B), which balance the negative charge of a half of a diprotonated decavanadate anion. The molecular structure of the cis-dioxidovanadium(V) complex consists of a distorted octahedral environment, in which the tpma acts as a tetradentate ligand. Precisely, the VO_2_ group is coordinated to tpma ligand by three pyridyl N atoms and one tertiary amine N atom, where O=V=O angle are 107.76(14) and 107.66(11)° for molecule A and B, respectively, V=O bond distances are in the 1.624(2)–1.631(2) Å range, V-Npyridyl bond lengths are between 2.090(3) and 2.277(2) Å, and bond distances of V-Namine are 2.250(2) and 2.258(2) Å for molecule A and B respectively. These values are consistent with similar cis-dioxo vanadium complex in octahedral geometry found in Cambridge Structural Database (CSD, Conquest) (Silva et al., 2011).

**FIGURE 2:**
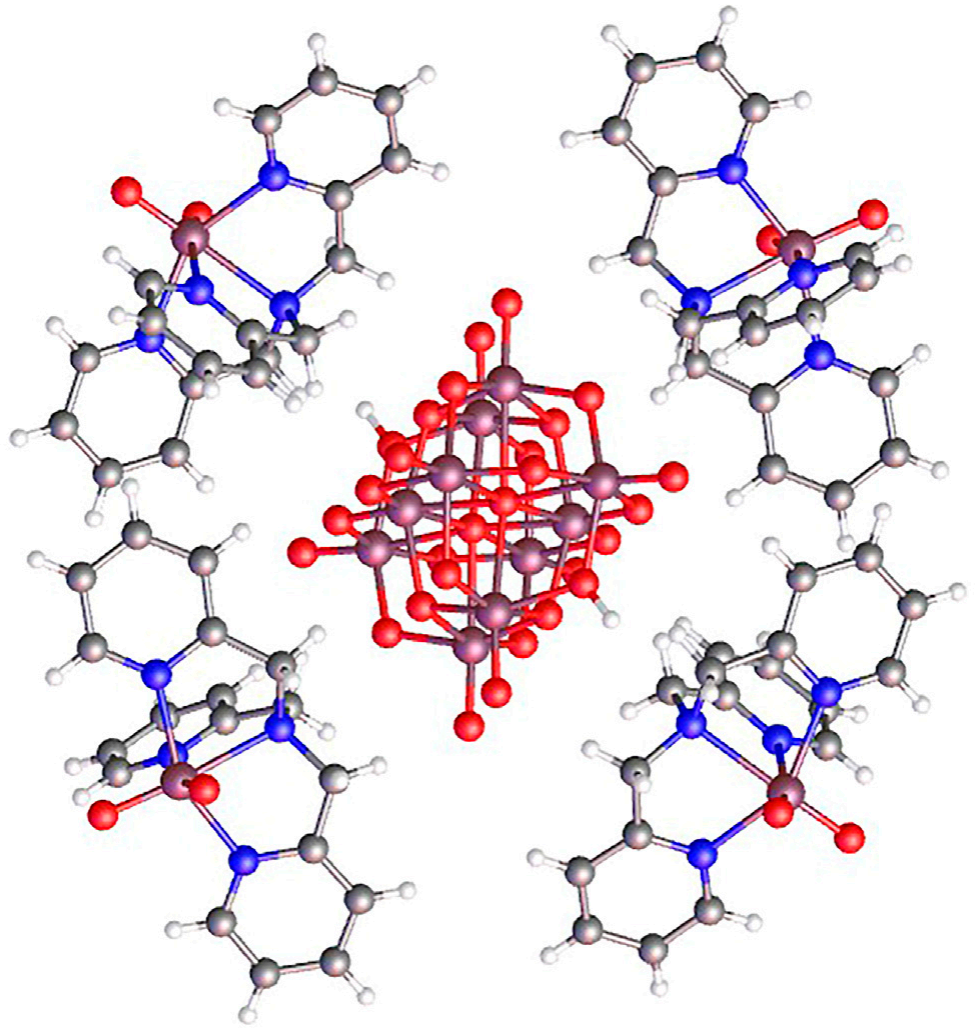
Expansion of asymmetric unit using OLEX program shows the tridimensional structure formed by a diprotonated decavanadate unit and four dioxidovanadium(V) complexes, water molecules were removed for clarity.

**FIGURE 3:**
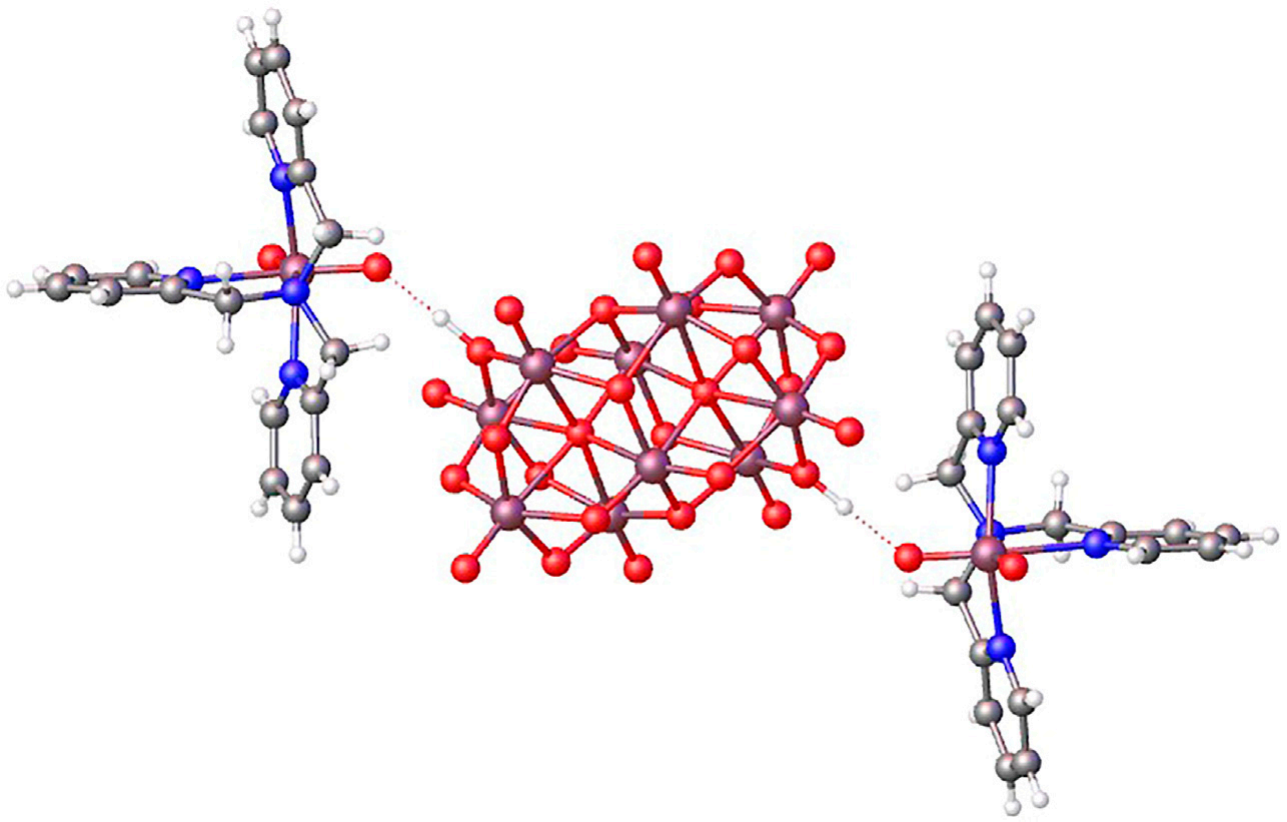
OLEX representation of hydrogen bonds connecting both species of V(V).

**FIGURE 4:**
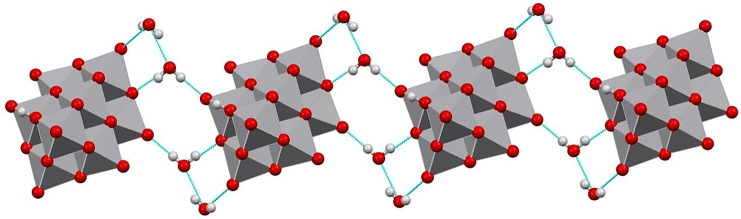
Mercury representation of a chain of decavanadate anions connected through water molecule forming rings R^3^_3_ (8) and R^4^_4_ (12).

**FIGURE 5:**
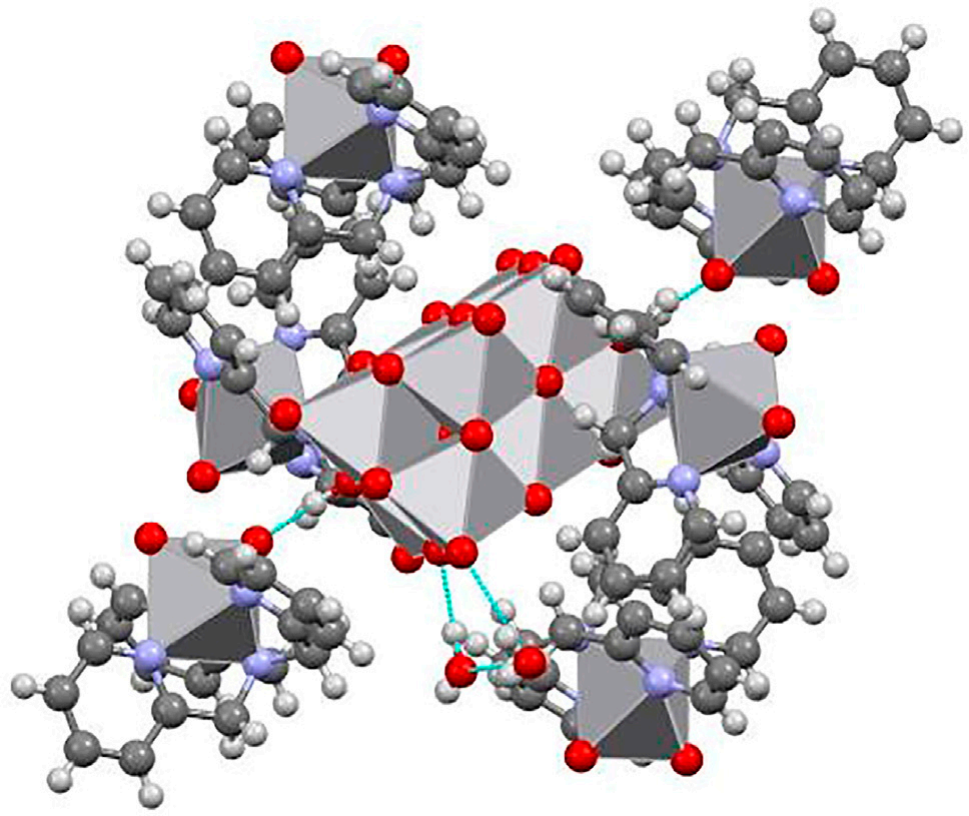
Supramolecular structure showing how a single diprotanated decavanadate is surrounded by six moieties of [VO_2_(tpma)]^+^.

The three-dimensional structure comprises decavanadate units, dioxidovanadium(V) complexes, and crystallization water molecules connected by hydrogen bonds (Figure 2). The two protons of decavanadate anion interact with two different [VO_2_(tpma)]^+^ cations through the O1A atom (Figure 3), whereas molecule B is not involved in the hydrogen network. Furthermore, crystallization water molecules interact between pairs of decavanadate clusters, thus stabilizing the supramolecular structure (Figure 4). Table 2 summarizes the hydrogen bonds for **Compound 1**. Figure 5 shows how the diprotanated decavanadate is surrounded by [VO_2_(tpma)] moieties.

**TABLE 2:** Hydrogen bonds.

D-H···A	D-H	H···A	D-H···A	Angle (°)
O10-H10···O1A	1.05	1.67	2.718 (3)	177.8
O2W-H2WA···O1W	0.82	2.14	2.906 (5)	155.0
O1W-H1WA···O6	0.87	2.05	2.915 (3)	170.1
O1W-H1WB···O3	0.95	1.96	2.901 (3)	168.5

### IR Spectrum

The FTIR spectrum of **Compound 1** is presented in Supplementary Figure S1; it shows the terminal V=Ot stretching bands within 1,000–900 cm^−1^. The strong V=Ot stretching vibration at 940 cm^-1^ has a shoulder at 970 cm^−1^ assigned to the cis V=O_2_ asymmetric band. These stretching vibrations agree with the terminal V=O groups present in related oxidovanadium(V) complexes (Biçer et al., 2017). The medium intensity bands at 840, 800, and 730 cm^−1^ corresponds to the asymmetric vibrations of the bonds (V–O_b_–V), where the oxygen atom bridge two vanadium ions, while the three bands at 570, 510, and 430 cm^−1^ reveal symmetric vibration modes of the bonds (V–O_b_–V), in agreement with other FTIR spectra reported previously for related compounds (Guilherme et al., 2010), (Sánchez-Lara et al., 2014; Sánchez-Lara et al., 2015; Sánchez-Lara et al., 2016; Sánchez-Lara et al., 2018; Sánchez-Lara et al., 2019; Sánchez-Lara et al., 2021), (García-García et al., 2021).

### ^51^V Nuclear Magnetic Resonance

Compound 1’s ^51^V NMR spectrum was measured in D_2_O at pH 6.8, and 25°C. It displays the typical ^51^V resonance signals assigned to the three different vanadium atoms of the [H_n_V_10_O_28_]^(6−n)−^ structure in the 2:2:1 population ratio, present at VC = −422 ppm, VB = −500 ppm and VA = −516 ppm and a signal at −509 corresponding to the dioxidovanadium complex (Supplementary Figure S2A). This signals corresponded to the unprotonated species [V_10_O_28_]^6−^ this suggests that the anion [H_2_V_10_O_28_]^−^ is subject to rapid deprotonation in aqueous solution [H_2_V_10_O_28_]^4−^ pKa = 4.17, (Sánchez-Lara et al., 2018). (Soares et al., 2007; Rehder, 2008; Aureliano and Crans, 2009; Rehder, 2015; Krivosudsky et al., 2019). The signals at = 572.52 and 577.06 ppm are due to mono (V_1_), divanadate (V_2_), and cyclic tetramer (V_4_), respectively (Rehder, 2008). This signals growth in a time dependent manner, while the signals of the decavanadate decrease (Supplementary Figure S2B).

### Theoretical Results

**FIGURE 6:**
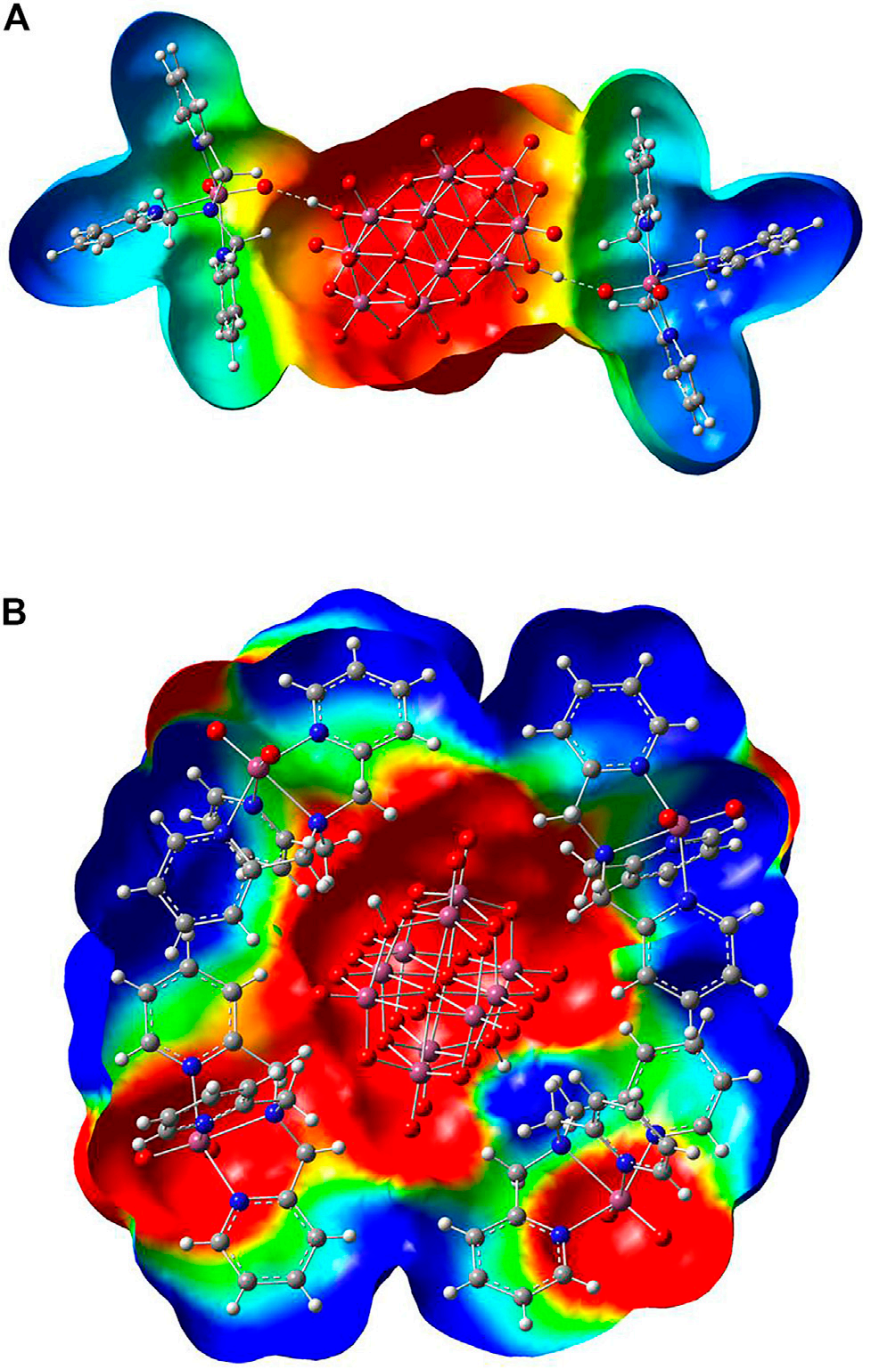
Molecular electrostatic potential map of **(A)** the representation of Compound 1 forming hydrogen bonds connecting both species of V(V), and **(B)** the asymmetric unit showing to the diprotonated decavanadate unit and four dioxidovanadium(V)-tpma moieties, calculated at the theory level mPW1PW91/6–31G(d)-LANL2DZ using ECP = LANL2DZ for the V atom.

Figure 6 shows the optimized structures of two representations of **Compound 1** mapped on the solid molecular electrostatic potential (MEP). In Figure 6A, the structure-forming hydrogen bonds connecting both species of V(V), decavanadate anion, and dioxidovanadium(V)-tpma cations are shown. Figure 6B presents the asymmetric unit showing the diprotonated decavanadate unit and four dioxidovanadium(V)-tpma moieties. These optimized structures and MEPs were calculated at the mPW1PW91/6–31G(d)-LANL2DZ level of theory using ECP for the V atom in the aqueous solvation phase. For MEP surfaces, the electrostatic potential was mapped on the total electronic density with isovalue = 0.004 a. u. The qualitative color code shows red regions for the nucleophilic zones (negative charge density), while blue regions indicate the electrophilic zones (deficient density charge). Yellow and green regions correspond to intermediate electron density zones. Figure 6A shows negative charge density on protonated decavanadate [H_2_V_10_O_28_]^4-^ anion indicating a nucleophilic zone, while the two dioxidovanadium(V)-tpma [VO_2_(tpma)]^+^ cations show positive charge indicating electrophilic zones. In addition, the H-bonds between one oxygen atom of the dioxidovanadium(V)-tpma and the hydrogen of the protonated decavanadate are located in intermediate regions of electron density, indicating important noncovalent interactions. Figure 6B shows the [H_2_V_10_O_28_]^4−^ anion with negative charge density surrounded by four [VO_2_(tpma)]^+^ cations. In the [VO_2_(tpma)]^+^ moieties, negative charge density regions are observed on dioxidovanadium and positive charge on tmpa groups. These electrophilic and nucleophilic regions are susceptible to interact with adjacent [VO_2_(tpma)]^+^ cations and water molecules, as shown in Figures 4, 5. The charge density distribution in both arrangements indicates the relevance of noncovalent interactions in the supramolecular structure.

**FIGURE 7:**
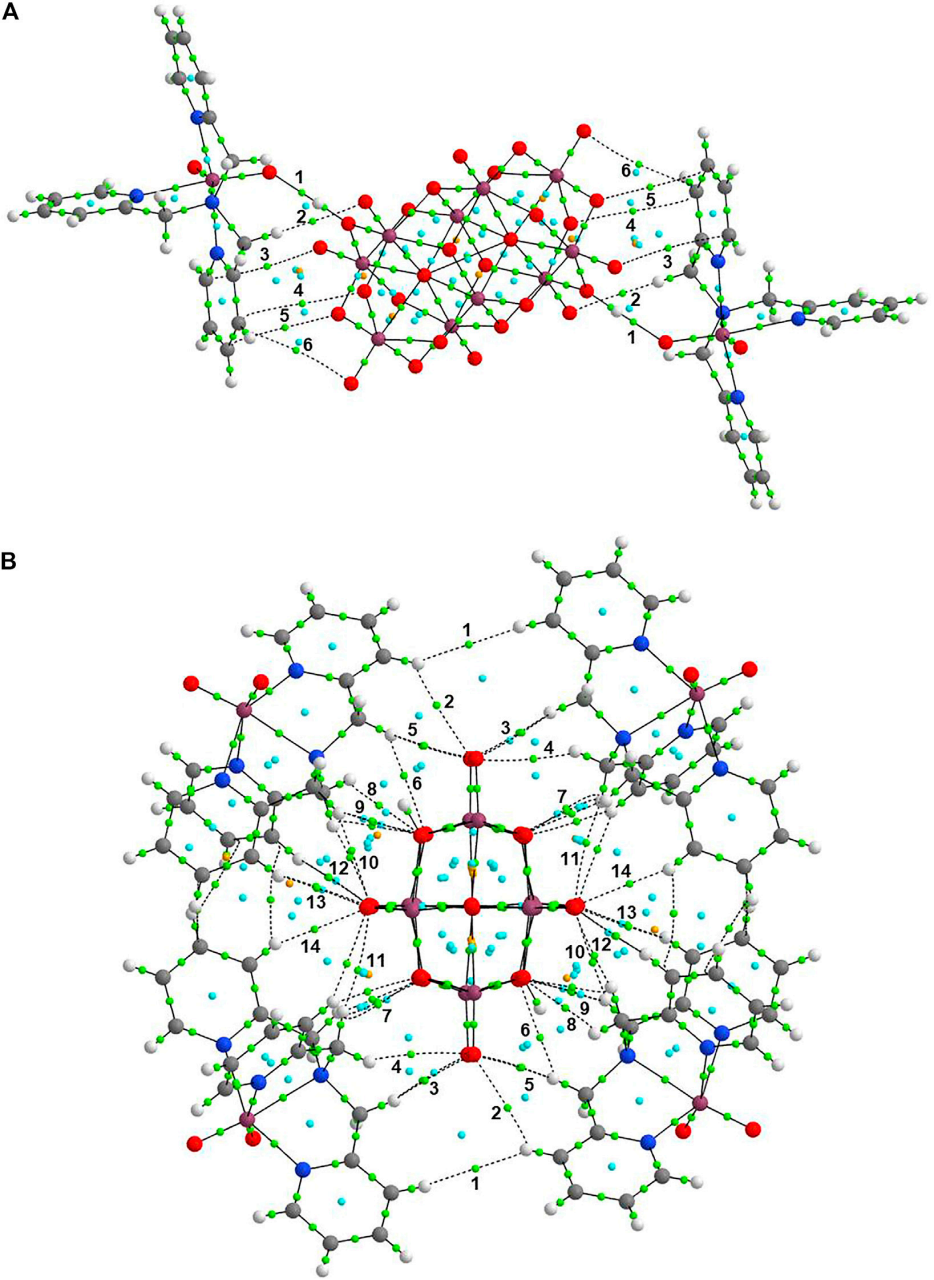
Molecular graphs of **(A)** the representation of **Compound 1** forming hydrogen bonds connecting both species of V(V), and **(B)** the asymmetric unit showing to the diprotonated decavanadate unit and four dioxidovanadium(V)-tpma moieties.

The main noncovalent bonds were analyzed by electron density, ρ(r), the Laplacian of density, ∇^2^ ρ(r), and the energy of interaction, E_H⋯Y_. The results are summarized in Table 3. In addition, Figure 7 shows the molecular graphs of both systems described in Figure 6A,B of Compound 1. In Figure 9, green dots represent bond critical points (BCPs), cyan dots represent ring critical points (RCPs), and orange dots represent cage critical points (CCPs). From the results for (a), it can be seen that the positive higher value of ∇^2^ ρ(r) for interaction 1 (see Figure 7) indicates that the noncovalent interaction is a hydrogen bond. The calculated interaction energy is 11.92 kcal mol^−1^ for this hydrogen bond connecting both V(V) species. Other interactions (2–5 in Figure 7A) were found in the system 1) with small interaction energies between 0.22–1.16 a. u. For system (b), a large number of weak interactions (1–14 in Figure 7B) were found surrounding the [H_2_V_10_O_28_]^4−^ anion, through oxygens atoms of the [H_2_V_10_O_28_]^4−^ with hydrogen atoms of tmpa moieties. All of them are of C-H⋯O type. The maximum value of ρ(r) was found for interaction 10 (C103-H104⋯O30) with the highest interaction energy of 2.60 kcal mol^−1^. Many ring points are also observed, which indicate the formation of stable rings in the structure. Also, some cage critical points are found between TPMA molecules and [H_2_V_10_O_28_]^4−^ anion, as well as in the central [H_2_V_10_O_28_]^4−^ anion.

**TABLE 3:** Topological parameters (in a.u.) and interaction energies E_H⋯Y_ (in kcal mol-1) of (a) the representation of Compound 1 forming hydrogen bonds connecting both species of V(V), and (b) the formula unit showing the diprotonated decavanadate and four dioxidovanadium(V)-tpma moieties.

Interaction	**(a)**			
	BCP	ρ(r)	∇^2^ρ(r)	E_H···Y_
1	O39-H40···O42	0.0451	0.1426	11.92
2	C48-H50···O26	0.0058	0.0234	1.16
3	O37···C58	0.0015	0.0063	0.22
4	O34···C52	0.0025	0.0095	0.41
5	O36···C54	0.0039	0.0144	0.72
6	O35···C52	0.0028	0.0101	0.44
**(b)**				
	**BCP**	**ρ(r)**	**∇^2^ρ(r)**	**E_H···Y_**
1	C181-H182···H96-C95	0.0011	0.0044	0.13
2	C95-H96···O13	0.0040	0.0172	0.72
3	C177-H178···O13	0.0049	0.0200	0.94
4	C201-H203···O13	0.0054	0.0218	0.75
5	C91-H92···O13	0.0064	0.0260	1.35
6	C91-H92···O27	0.0054	0.0244	1.16
7	C189-H191···O40	0.0088	0.0346	2.04
8	C103-H105···O9	0.0073	0.0294	1.54
9	C115-H117···O35	0.0084	0.0337	1.88
10	C103-H104···O30	0.0110	0.0409	2.60
11	C76-H77···O39	0.0065	0.0271	1.38
12	C107-H108···O30	0.0049	0.0217	0.97
13	C119-H120···O34	0.0049	0.0216	0.97
14	C64-H65···O30	0.0054	0.0220	1.07

Analysis of Hirshfeld surfaces (HS) from a crystal structure is a valuable tool for calculating and visualizing intermolecular interactions. HS are generated based on the charge density distribution calculated as the sum of hard spheres electron densities (Hirshfeld, 1977). In the HS, d_i_ indicates the distance from the surface to the nearest nucleus inside the surface, and d_e_ is the distance from the surface to the nearest nucleus outside the surface. The ratio between d_i_ and d_e_ and the van der Waals radii of the atoms define the function d_norm_, named normalized contact distance (Spackman and Byrom, 1997). The red spots in the HS indicate that the sum of distances d_i_ and d_e_ is shorter than the sum of the van der Waals radii (negative d_norm_), specifying closest contacts. The blue regions indicate more extended contacts than the sum of the van der Waals radii (positive d_norm_). Furthermore, the white regions indicate contacts close to van der Waals radii (d_norm_ equal to zero). The fingerprint plot is the 2D representation of HS in terms of d_i_ versus d_e_. The fingerprint plot provides the contributions of the most representative intermolecular interactions to the total surface (Parkin et al., 2007). In the fingerprint plot, the color of each point is a function of the fraction of the total surface points. The color ranges from blue (few points contributing to the surface) to green-yellow until red (many points contributing to the surface). Also, characteristic spikes on the fingerprint plots indicate particular interactions, for example, O ⋯ H/H ⋯ O, H ⋯ H, C ⋯ H, or halogen ⋯ H. Fingerprint plots are unique for each molecular crystal (Spackman and McKinnon, 2002).

**FIGURE 8:**
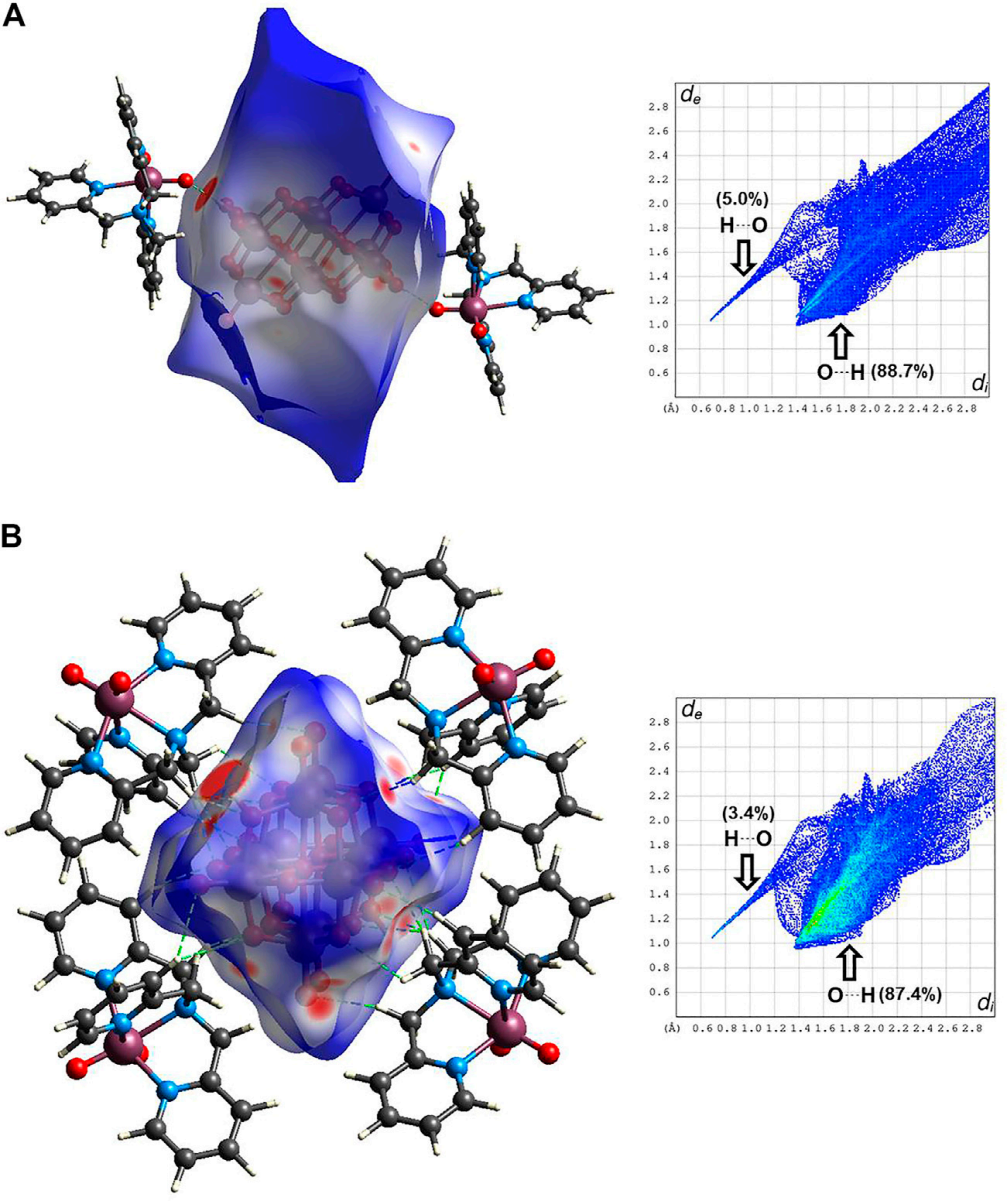
Hirshfeld surfaces (left) and fingerprint plots of noncovalent interactions (right) mapped with d_norm_ parameter of **(A)** the representation of **Compound 1** forming hydrogen bonds connecting both species of V(V), and **(B)** the asymmetric unit showing to the diprotonated decavanadate unit and four dioxidovanadium(V)-tpma moieties.

The HS and the fingerprint plot of **Compound 1** were mapped with the function d_norm_ **,** as shown in Figure 8. From the results, it is observed for system (a) that the most prominent red spots on the Hirshfeld surface are due to the hydrogen bonds between O-H inside the HS and the oxygen of the [VO_2_(tpma)]^+^, indicating strong hydrogen bonds with an interatomic distance of 1.736 Å and 175.68°. These are indicated with dashed green lines in Figure 8A. Other minor red spots are located on the surface, indicating the close intermolecular interactions of the oxygen atoms of [H_2_V_10_O_28_]^4−^ anion with hydrogen atoms outside the HS. In its corresponding fingerprint plot, the hydrogen bonds OVO ⋯ H-O connecting both species of V(V) has a contribution of 5.0% of the HS, while the close intermolecular interactions O ⋯ H of [H_2_V_10_O_28_]^4-^ anion and H outside the HS contribute in 88.7%, as indicated in Figure 8A. Other interactions with minor contributions are O ⋯ C (3.4%) and H ⋯ H (2.2%).

For system (b), all the red spots on the HS correspond to close intermolecular interactions between oxygen atoms of [H_2_V_10_O_28_]^4−^ acting as acceptors of the hydrogen atoms of the tpma moieties. This interaction contributes 87.4% in the fingerprint plot, corresponding to the close intermolecular interactions indicated with dashed green lines in Figure 8B. On the other hand, the hydrogen bond interactions, where the protonated oxygens of the [H_2_V_10_O_28_]^4−^ act as a donor, contribute only with the 3.4% of the HS. Other non-covalent interactions with minor contributions in the system (b) are O ⋯ C (4.0%), H ⋯ H (3.0%), and O ⋯ O (1.2%).

### Molecular Docking Analysis

**FIGURE 9:**
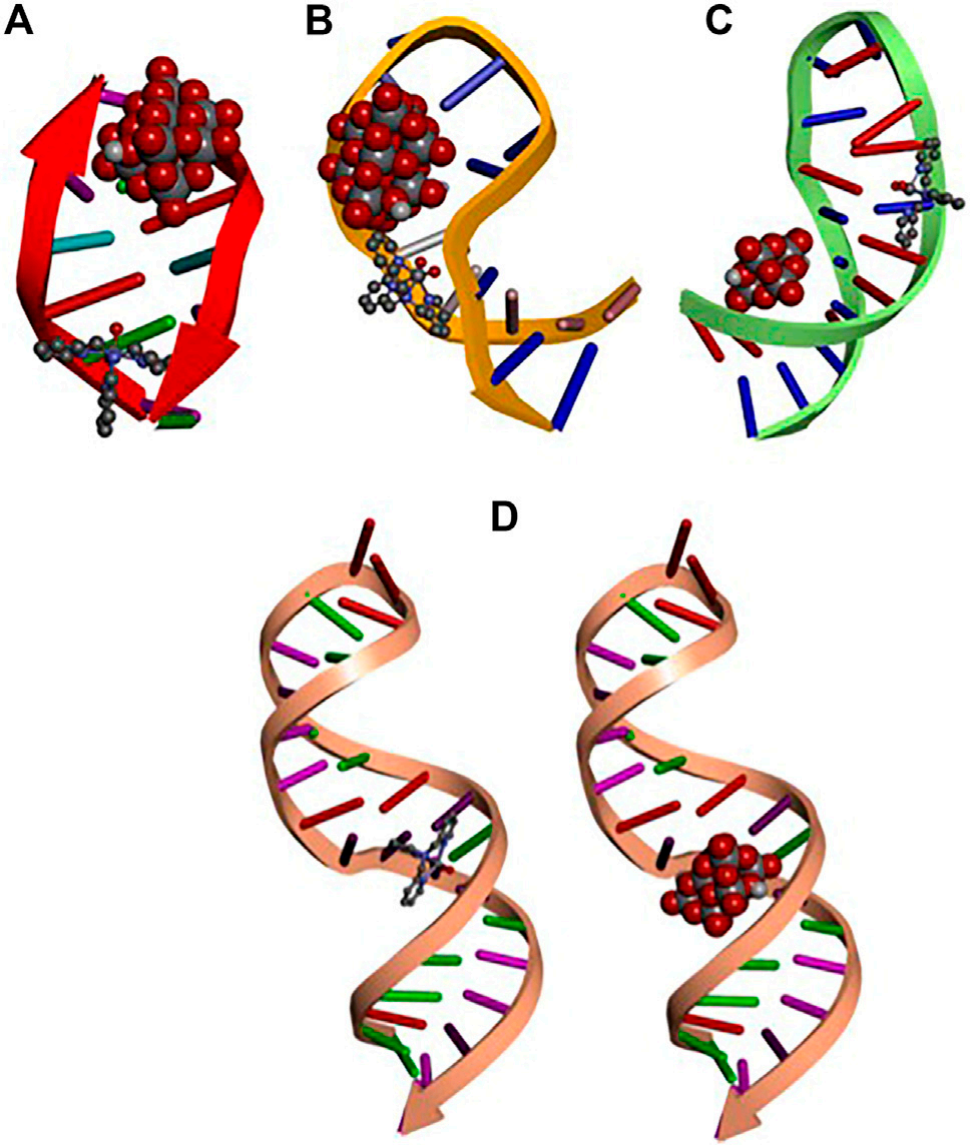
Docked structures of top molecular pose for: **(A)** DNA **(B)** pre-miR-21 **(C)** l7-miRNA **(D)** lncRNA with diprotonated decavanadate shown in CPK display style and [VO_2_(tpma)]^+^ shown in scaled ball and stick style.

The docked binding energies and the interaction with the different targets corresponding to the top molecular pose (lowest energy) for Tris(2-Pyridylmethyl)amineV(O)_2_, cation [VO_2_(tpma)]^+^, and decavanadate are shown in Table 4; Figure 9. The results obtained in the interaction between decavanadate and diprotonated decavanadate with the different targets remain the same in the interactions with pre-miR-21(PDB ID: 2MNC). However, the binding energies are slightly improved in the diprotonated decavanadate when interacting with DNA (PDB ID:151D), l7-miRNA (PDB ID:2JVX), and lncRNA (PDB ID: 6PK9) by 1 kcal/mol. In all the structures, the number of hydrogen bonds are between 1 and 3.

**TABLE 4:** Molecular docking results. Binding energies for the deprotonated decavanadate anion best molecular poses with DNA tRNA and lncRNA.

Compound	Target	Binding energies (kcal/mol)	H Bond	Interactions
Diprotonated Decavanadate	151D (DNA)	−10.88	4	G2, A3, T4, C5, G6
	2MNC (pre-miR-21)	−*8.91*	2	C8, G10, C9, C21, A20
		−*8.67*	3	G13, A14, C21, G13, A14, C17
	2JXV (l7-miRNA)	−10.27	1	U6,A7,G8,U9,U24, U25
		−9.77	1	A3,G4,G5,A7,U25,C26,U27
	6PK9 (lncrna)	−9.77	2	G2, A3, G4, U16, C17
		−9.64	4	A3, G4, G5, C15, U16
Decavanadate	151D (DNA)	−10.1	3	G2, A3, T4, C5, G6
	2MNC (pre-miR-21)	−8.92	2	C8, G10, C9, C21, A20
		−8.67	3	G13, A14, C21, G13, A14, C17
	2JXV (l7-miRNA)	−8.66	1	G8, U24, U25, U9, C26, A7I6
		−8.58	5	G11, G12, G19,A20, G15
	6PK9 (lncrna)	−8.48	1	A3, G1, G2, C17, U16
		−8.39	3	G4,G6,C15, G5
	151D (DNA)	−6.85	1	G2,A3,C5,G6
	2MNC (pre-miR-21)	−6.39	1	G10,U11,U12,G13,A20,C21
		−6.07	1	C8, C9, A20,C21,G22,G23
	2JXV (l7-miRNA)	−6.17	1	U6,A7,G8,U9,U24
		−5.94	1	G11, G12, U13
	6PK9 (lncrna)	−6.05	1	U7, A8, G9
		−5.85	1	A3,G4,G5,G6

On the other hand, when docking was carried out between the targets and the [VO_2_(tpma)]^+^ the median binding energies were found in −6.07 kcal/mol, the interactions involved mainly comprise of van der walls, π-π, and H bond interactions.

**FIGURE 10:**
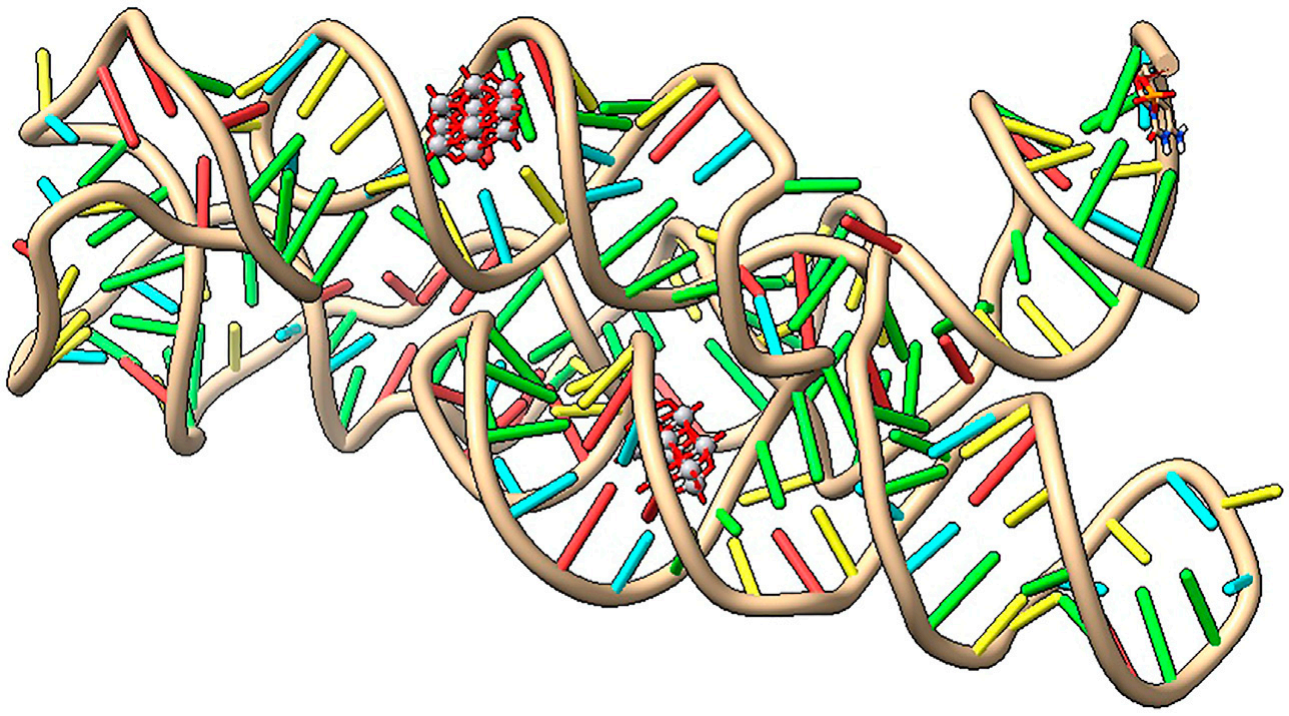
Interaction of decavanadate with the riboswitch (3DIS) visualized with UCSF Chimera X program, (2021).

From Figure 9 can be seen that the ligands decavanadate and [VO_2_(tpma)]^+^ interact in a similar position with the targets pre-miR-21 and l7-miRNA while in the DNA and *lnc*RNA structures, the ligands interact in different positions with could lead to a double interaction from the Tris(2-Pyridylmethylamine)V(O)_2_ complex, the anionic and the cationic moieties interacting with the same target but in different positions that could improve their pharmacological properties. Figure 10 shows the interaction of decavanadate with a riboswitch considered a test molecule for mRNA (PDB ID: 3DIS) (Serganov et al., 2008). Table 5 gives the energies of interaction for the best poses of decavanadate in the structure of 3DIS.

**TABLE 5:** Binding energies for best molecular poses of decavanadate in complex with Riboswitch (PDB ID:3DIS).

Compound	Target	Binding energies (kcal/mol)	Interactions
Decavanadate	riboswitch	−11.81	H bond (2) G118, A120/C132, A133, A120, U119, A135, G118, G117
		−10.67	H bond (3) G90, G101, C102/U93, C100, G101, C102, G92, A91, G90, A89

## Discussion

Because the global incidence of cancer increases every year, as does resistance to currently available chemotherapeutic medications like cisplatin, one of the pharmaceutical industry’s top concerns today is developing more effective cancer treatments (Aureliano 2017).

Vanadium is mainly found in the form of vanadate H_2_VO^4−^, which is most likely involved in the regulation of phosphate-dependent processes like metabolic pathways involving phosphatases and kinases and phosphate metabolism in general due to its structural and chemical similarity to phosphate. However, at pH values less than neutral, a complicated mixture occurs, mainly formed of decavanadate anions that can exist in six distinct protonation states depending on the pH (Crans et al., 2014; Crans et al., 2017).

Among the broad family of polioxidovanadates, the ion decavanadate [H_n_V_10_O_28_]^−y^ is one of the most common clusters in the region of acidic pH. In the last years, the study of noncovalent interactions emerging from the Coulombic attraction between the decavanadate ion and certain biologically important cations has generated increasing attention. Investigation of these interactions could provide greater insight into these compounds’ biological mechanisms of action. The level of protonation of the [V_10_O_28_]^6−^, as well as the type of the cation and solvent, are all important for generating monomers, dimers, and supramolecular assemblies in one, two, or three dimensions (Ferreira da Silva et al., 2003), (Kasuga et al., 2009), (Sánchez-Lara et al., 2014), (Amanachi et al. 2018). The decavanadate anion, contraiones (which can be of variable size and nature, both inorganic and organic), and a certain number of molecules of crystallization water support interactions of van der Waals and hydrogen bonds that modulate the supramolecular structure in the majority of compounds of this nature. The decavanadate then works as a species acceptor of protons from the remainder of the molecule’s atoms, allowing NH···O_deca_, OH···O_deca_, and CH···O_deca_ hydrogen bonds, acting as a molecular engineering cement.

Decavanadate, which is easily identifiable by its distinctive yellow-orange color, is formed from metavanadate in the presence of an acid and consumes protons, whereas decavanadate hydrolysis generates protons. The qualitative manifestation of this reaction is the reversible shift in the hue of the solution from yellow decavanadate-enriched to clear oligomeric vanadates. While decavanadate is not the thermodynamically stable form at neutral pH values, it is kinetically stable for up to 48 h at neutral pH, and depending on the conditions.

Organic ligands may also aid in modifying vanadium’s bioavailability, transport, and targeting processes; as a result, coordination compounds containing vanadium have been increasingly popular in recent years due to their potential for treating diabetes, cancer, leishmaniasis, and HIV (Rehder 2008; Rehder, 2012; Rehder, 2015; Rehder, 2020). As a result, various researchers have discovered decavanadates as prospective anticancer drugs with promising tumor growth inhibition outcomes. Although the anticancer activity of decavanadate is less well known, it is thought to be connected to its inhibition of a range of enzymes, including alkaline phosphatases, ectonucleotidases, and P-type ATPases (McLauchlan et al., 2015; Aureliano 2017; Aureliano et al., 2022). A recent review indicates the activity of decavanadates in cancer, bacteria, and viruses, including apoptosis, cell cycle arrest, interference with ions transport system, inhibition of mRNA synthesis, cell morphology changes, changes in metabolic pathways, phosphorylase enzyme inhibition and cell signaling, formation of reactive oxygen species, lipid peroxidation, inhibition of viral mRNA polymerase, inhibition of virus binding to the host cell, penetration, and interaction with virus protein cages (Aureliano et al., 2021).

One of the first papers on decavanadate compounds with anticancer activity was reported by Zhai et al., in 2009. The compound Na_4_Co(H_2_O)_6_ [V_10_O_28_]•18H_2_O inhibits the proliferation of human liver cancer (SMMC-7721) and ovarian cancer (SK-OV-3) cell lines *in vitro* more effectively than the currently used antitumoral agent 5-fluorouracil, which can reduce liver tumor weight in rats *in vivo* (Zhai et al., 2009). In 2010, Li et al. developed two decavanadates with organic ligands that inhibited human lung development (A549) and murine leukemia (Li et al., 2010). A slew of other papers on decavanadate compounds with anticancer potential have appeared (Kioseoglou et al., 2013; Cheng et al., 2018; Gu et al., 2020; Louati et al., 2021; Ksiksi et al., 2021a; 2021b).

On the other hand, studies on Tris(2-pyridylmethyl)amine as an osteosarcoma (OS) antitumor agent is linked to higher miRNAs and proteins involved in several signaling pathways. This syndrome promotes tumor survival and resistance and plays an important role in carcinogenesis. TPMA interacts with both the 5′ and 3′ strands of miRNA via van der Walls interactions (Palmeira-Mello et al., 2020).

Apart from the well-known isulinomimetic activity, Bis (ethylmaltolato) oxidovanadium (IV) BEOV reduced tau hyperphosphorylation by inhibiting PTP1B expression, which increased IR/IRS-1/PI3K/Akt signaling and hindered GSK3 activity. BEOV also increased the breakdown and clearance of A deposits and tau aggregates by promoting autophagolysosomal fusion and restoring autophagic flow. These findings established BEOV as a promising therapeutic medication for treating Alzheimer’s disease (He et al., 2020). Also, when combined with oncolytic viruses (OVs), vanadium compounds can provide a significant therapeutic benefit in CT26WT and other aggressive, treatment-resistant murine tumor models, and resulting in increased antitumor T cell responses mediated by the induction of type II Interferon-like response in infected cancer cells (Selman et al., 2018). Vanadium constitutes a well-known phosphate analog. Hence, its study offers possibilities to design promising vanadium-containing binders to SARS-CoV-2. Since vanadium nuclei have favorable physicochemical properties, organic vanadium complexes could be used as drugs and diagnostic tools for early infection detection in patients (Scior et al., 2021). Recently, DFT calculations and molecular docking were performed on BSA (transport protein), 6M03 (Covid-19 major protease), 6M71 (SARS-CoV–2 RNA-dependent polymerase), and 6YI3 (N-terminal RNA-binding domain of SARS-CoV–2 nucleocapsid phosphoprotein). In addition, several vanadium complexes appear to have promising antiviral possibilities (Vlasiou and Pafti 2021). Since vanadium compounds may be new treatments for diabetes, cancer, atherosclerosis, and other disorders, they have also shown a broad range of anti-RNA virus activity, making them promising antiviral agents. Given these advantages, the link between COVID-19 and diabetes and the antiviral, antihyperglycemic, insulin-enhancing, and anti-inflammatory properties of vanadium compounds may be considered adjuncts to COVID-19 treatment (Semiz 2021).

Small non-protein-coding RNAs and mRNA sequences play a central role in cellular regulatory processes and are involved in virtually every aspect of maintaining and transmitting genetic information. Thus, as a working hypothesis, we expand the interaction of decavanadate to RNA as a target molecule (García-García et al., 2021). Docking studies on the interaction of decavanadate anion with various microRNAs (miRNAs) and DNA molecules were evaluated for the putative anticarcinogenic activity. MiRNAs are small non-coding RNA molecules that affect a wide range of target genes. They range in length from 19 to 25 nucleotides. Around 30% of human genes are controlled by miRNAs, half of which are tumor-related. Differentiation, proliferation, energy consumption, and immune response are just some of the many activities they perform. Apoptosis, invasion, and metastasis have all been linked to the activity of miRNAs (Noda 2012) (Si et al., 2019). However, upregulation of miR-21 and lncRNA (long non-coding RNA) has been linked to various cancers, such as malignant B-cell lymphoma and breast cancer (Feng and Tsao, 2016; Chi et al., 2019; Zhao et al., 2021; Sheykhhasan et al., 2021). Let-7 miRNA, on the other hand, is known as the “keeper of differentiation” and has been discovered as possible cancer and immune response therapeutic agent (Gilles and Slack, 2018; Chirshev et al., 2019). Our first findings indicate that decavanadate has a high affinity for miRNA segments (García-Garcia et al., 2021). Here we show that the interaction of decavanadate and Vdioxido-tpma complex can interact with these target molecules; thus, experimental efforts should be carried out to explore this possibility.

Vanadium compounds can also be used in a wide variety of catalytic processes, and many research papers and reviews on specific themes of vanadium catalysts have lately been published (Huang et al., 2020; Sutradhar et al., 2020). Decavanadates have a wide range of applications in biology; however, several papers have shown that they can also work as a catalyst (Hill and Bouchar, 1985; Steens et al., 2010; Foster et al., 2011) and recently for the photochemical oxidation of water to dioxygen (Buvailo et al., 2020). Redox centers, such as decavanadate units can build bifunctional platforms using transition metals. Thus-obtained nanoclusters have the potential to serve as effective bifunctional catalysts for the recovery or destruction of CO_2_ and chemical warfare agents (CWAs) at ambient temperatures (Huang et al., 2021). On the other hand, Vanadium compounds will continue to offer exciting growth opportunities in various industries.

## Conclusion

A new diprotonated decavanadate was synthesized and characterized by FTIR spectroscopy, ^51^Vanadium NMR, and single-crystal x-ray diffraction. The compound crystallized in the P21/c space group of the monoclinic system. The well-known tripodal ligand TPMA was introduced to explore the possibility that a protonated TPMA molecule could serve as a counterion of the decavanadate anion to serve as a model for histidine binding sites in proteins (Aureliano et al., 2022). However, the result was unexpected since the TPMA molecule coordinated to dioxidovanadium (V). Four of these units surrounded a diprotonated decavanadate anion. Also, hydrogen bonds bound two other dioxidovanadium (V) moieties in apical positions. In addition, two non-innocent water molecules allow the formation of a linear chain of decavanadate anions.

Noncovalent interactions have recently received much attention because they are self-assembled and provide supramolecular stability. Hydrogen bonding plays a vital role in crystals because of its highly directive nature, strong and specific interactions. These interactions exist in polyoxidovanadates groups, and the cohesion of these compounds is ensured by hydrogen bonds and van der Waals interactions; for that reason, the compound was analyzed theoretically; the molecular electrostatic potential indicates the electronic density distribution and the reactive sites on the compound. In solid-state, the protection of the central part of the decavanadate anion is observed surrounded by the dioxidovanadium(V)-TMPA units. Interestingly, in solution, the compound behaves as the TPMA was not coordinated. Instead, the dioxide moieties were rearranged to form the cyclo-tetravanadate or other small vanadate species. This finding points to the high stability of the cyclo-tetravanadate anion since TPMA is considered a strong chelator. Therefore, the weak noncovalent interactions, although necessary in the solid-state as evidenced in the Hirshfeld analysis and the AIM calculations, *i.e.,* hydrogen bonds with interaction energies of 11.92 kcal mol^−1^, do not appear to hold the molecule in water solution. Regardless of the speciation, the compound provides three components that have proved helpful as antitumor agents.

The presence of micro RNA, particularly in cancer cells, prompt us to investigate the interaction with these small molecules in which not all the bases are involved in intra-strand hydrogen bonds and therefore point towards a more exposed hydrophilic environment. Therefore, molecular docking studies using small RNA molecules were performed to test the hypothesis that decavanadate’s anticancer activity could be attributed to its interaction with miRNA molecules. The results obtained in the interactions between decavanadate and diprotonated decavanadate with the pre-miR-21 RNA, even though the interactions remain the same, yet the binding energies are slightly improved in the diprotonated decavanadate by 1 kcal/mol. As for the interaction with RNA and DNA molecules, it indicates an excellent possibility to induce brakes due to ROS generation as reduction of vanadium(V) to vanadium (IV) will take place in the cytosol, as ascorbic acid and reduced glutathione will react with vanadium species (Levina and Lay 2017; Costa-Pessoa and Correia 2021; Sciortino et al., 2021; Sanna et al., 2021; Sanna et al., 2021; Aureliano et al., 2021). Our recent findings of decavanadate interacting with bases such as cytosine, DMAP, and 2-aminopyridine, point to a high affinity of decavanadate to form stable hydrogen bonds with protonated nitrogenous bases or with water molecules serving as a bridge. It is important to mention that although we consider RNA and DNA highly negatively charged, they are permanently neutralized by cations interacting mainly with the polyphosphate chain, thus, allowing the nucleobases to interact directly with other molecules such as the proposed decavanadate. Notably, the energies of interaction with the decavanadate moiety are in the range of well-known anticancer drugs, suggesting that the repulsions with the sugar-phosphate backbone are not a critical fact. Also, the interactions between the RNA structures with the decavanadate involve interactions located in pockets around amino or imino groups in which the phosphate groups are not in proximity (Auffinger et al., 2004). The exact mechanism of how the decavanadate encountered RNAs is still unexplored. Thus, it is worthwhile to pursue this interaction experimentally, as this study suggests. Therefore the compound could be considered a metallo-prodrug that can be tested in several cancer cell lines. Since cancer cells exhibit increased levels of translation of mRNA to meet tumor growth requirements, it will be promising to explore the interactions of polyoxidovanadates with mRNA as suggested by the interaction of decavanadate with the riboswitch 3DIS (Ortega et al., 2021). Therefore, novel and captivating decavanadate chemistry will be developed in the near future.

## Data Availability

Selected crystal data and details are shown in Table 1 and Supplementary Table S1,S2. The CCDC number is 2124254 (Compound 1). Additional crystallographic data for this paper is presented in the Supplementary Material. You can obtain complete data free of charge from http://www.ccdc.cam.ac.uk/conts/re-trieving.html (or CCDC, Cambridge, United Kingdom; e-mail: deposit@ccdc.cam.ac.uk).
