# Supplementary material for: Tris(2-Pyridylmethylamine)V(O)2 Complexes as Counter Ions of Diprotonated Decavanadate Anion: Potential Antineoplastic Activity
Source: Front Chem. 2022 Feb 16;10:830511. doi: 10.3389/fchem.2022.830511 (PMC8888438; doi:10.3389/fchem.2022.830511)

Supplementary Material

Tris(2-Pyridylmethylamine)$V(O)_2$ complexes as counter ions of diprotonated decavanadate anion: Potential antineoplastic activity

Nidia D. Corona-Motolinia[1], Beatriz Martínez-Valencia[1], Lisset Noriega[2], Brenda L. Sánchez-Gaytán[1], Francisco J. Meléndez-Bustamante[2], Amalia García-García[3], Duane Choquesillo-Lazarte[4], Antonio Rodríguez-Diéguez[3], María Eugenia Castro[1*] and Enrique González-Vergara[1*]

[1]Centro de Química del Instituto de Ciencias, Benemérita Universidad Autónoma de Puebla, 18 sur and Av. San Claudio, Col. San Manuel, C.P. 72570 Puebla, Mexico

[2]Facultad de Ciencias Químicas, Benemérita Universidad Autónoma de Puebla, 18 sur and Av. San Claudio, Col. San Manuel, C.P. 72570 Puebla, Mexico

[3]Departamento de Química Inorgánica, Facultad de Ciencias, Universidad de Granada, Av. Fuente Nueva S/N, 18071 Granada, Spain

[4]Laboratorio de Estudios Cristalográficos, IACT, CSIC-Universidad de Granada, Av. de las Palmeras 4, 18100 Armilla, Granada, Spain

Figure S1. FTIR spectrum of Compound 1.

Figure S2A and S2B. ^{51}VNMR spectrum of Compound 1

Table S1. Bond distances for [VO2(tpma)]4[H2V10O28]•4H2O (1).

Table S2. Bond valence calculations for oxygen atoms in [H2V10O28]4- for Compound 1.

Checkcif of the deposited structure.

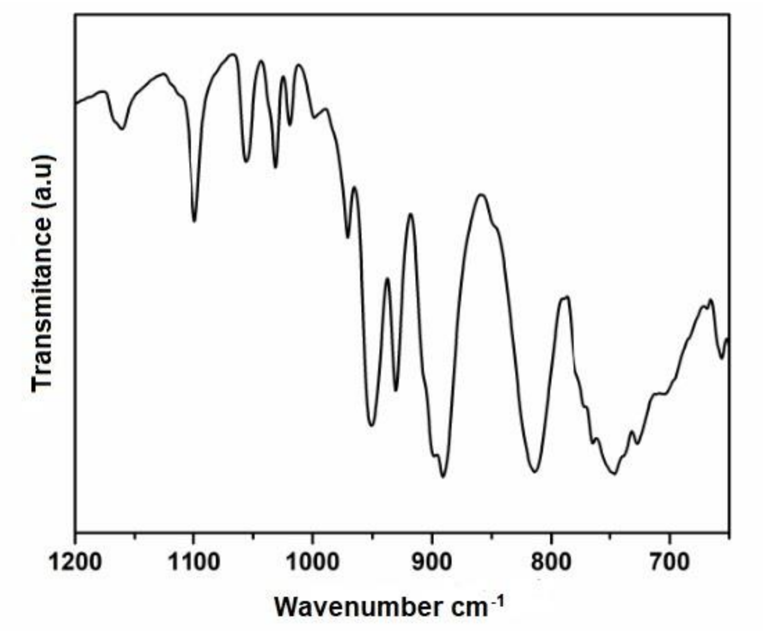

Figure S1. FTIR Spectrum of Compound 1 from 1000-400 cm^{-1} in the region of decavanadate vibrations V=O$_t$ and V-O$_b$-V.

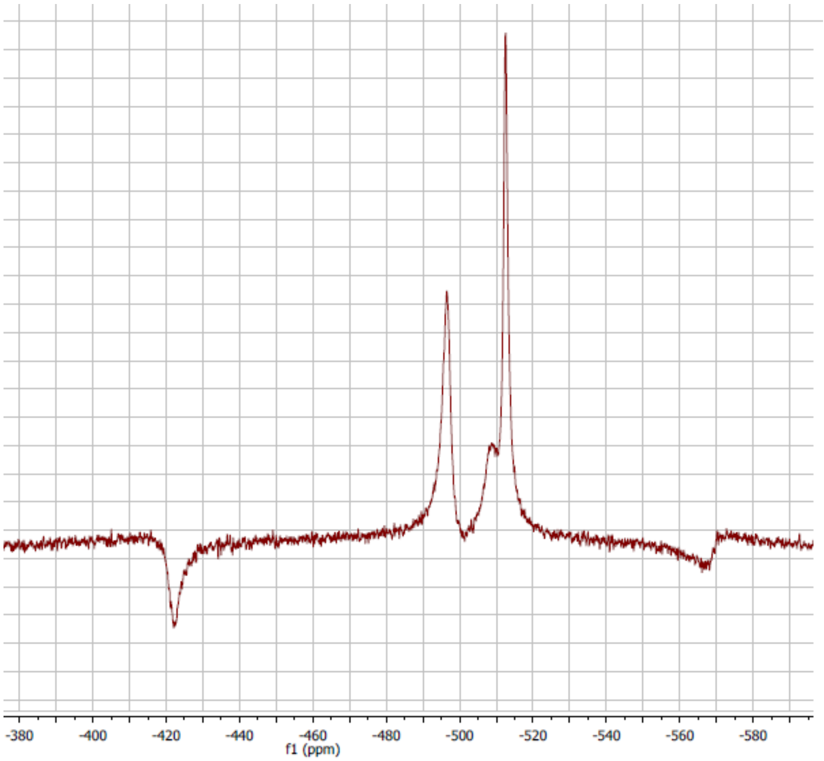

Figure S2A. ^{51}V **NMR of Compound 1 dissolved in** D_2O **(pH 6.8) at 25°C. Typical signals of decavanadate and a peak at -509 that could be assigned to the** $[VO_2(tpma)]$ **complex.**

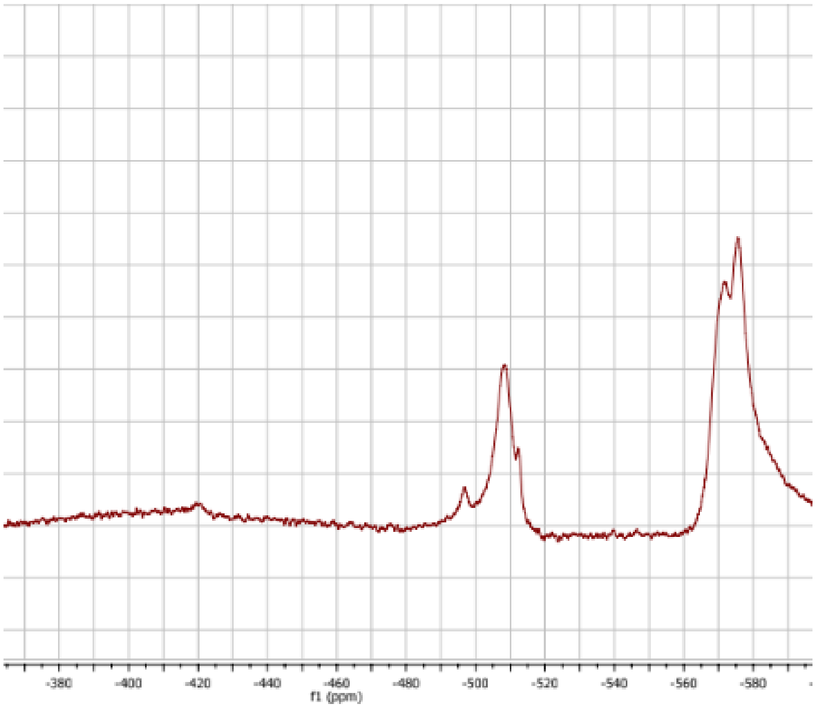

Figure S2B. After one week ^{51}V **NMR of Compound 1 dissolved in D2O (pH 6.8) at 25°C. Typical signals of decavanadate have decreased , the peaks of V1 and V4 have increased and a peak at -509 that could be assigned to the** $[VO_2(tpma)]$ **complex remained.**

Table S1. Bond distances for $[VO_2(tpma)]_4[H_2V_{10}O_{28}]\cdot 4H_2O$ (1)

Bond	Length (Å)	Bond	Length (Å)
V1-V2	3.0983(6)	N3A-C12A	1.342(4)
V1-V3(i)	3.0171(6)	N4A-C14A	1.335(4)
V1-V4	3.1053(6)	N4A-C18A	1.346(4)
V2-V5	3.0586(6)	C1A-C2A	1.487(5)
V3-V4	3.0939(7)	C2A-C3A	1.387(5)
V3-V5	3.0988(6)	C3A-C4A	1.372(6)
V4-V5	3.0587(6)	C4A-C5A	1.368(7)
V1-O1	2.2268(17)	C5A-C6A	1.370(6)
V1-O2	2.0670(17)	C7A-C8A	1.490(5)
V1-O3	1.7576(18)	C8A-C9A	1.382(5)
V1-O6	1.6111(18)	C9A-C10A	1.379(6)
V1-O10	1.9463(18)	C10A-C11A	1.359(7)
V1-O14	1.9203(17)	C11A-C12A	1.386(6)
V2-O1(i)	2.1390(16)	C13A-C14A	1.493(4)
V2-O1	2.1147(16)	C14A-C15A	1.389(4)
V2-O2	1.9162(17)	C15A-C16A	1.367(5)
V2-O5	1.6873(17)	C16A-C17A	1.370(6)
V2-O9	1.6876(17)	C17A-C18A	1.371(5)
V2-O14(i)	1.9465(17)	V1B-O1B	1.630(2)
V3-O1	2.2884(17)	V1B-O2B	1.624(2)
V3-O2(i)	1.9343(17)	V1B-N1B	2.258(2)
V3-O4	1.7987(18)	V1B-N2B	2.111(3)
V3-O8	1.6063(18)	V1B-N3B	2.277(2)
V3-O12	1.9045(17)	V1B-N4B	2.123(2)
V3-O14(i)	2.0186(17)	N1B-C1B	1.490(4)
V4-O1	2.3271(16)	N1B-C7B	1.485(4)
V4-O3	1.9436(18)	N1B-C13B	1.486(4)
V4-O4	1.8657(18)	N2B-C2B	1.354(4)
V4-O5(i)	2.0867(18)	N2B-C6B	1.342(4)
V4-O7	1.6099(19)	N3B-C8B	1.340(3)
V4-O11	1.7798(18)	N3B-C12B	1.341(4)
V5-O1	2.3064(16)	N4B-C14B	1.351(3)
V5-O9	2.0398(18)	N4B-C18B	1.339(4)
V5-O10	1.9998(18)	C1B-C2B	1.495(4)
V5-O11	1.8649(18)	C2B-C3B	1.383(4)
V5-O12	1.7716(18)	C3B-C4B	1.375(5)
V5-O13	1.6033(19)	C4B-C5B	1.379(5)
V1A-O1A	1.631(2)	C5B-C6B	1.377(5)
V1A-O2A	1.624(2)	C7B-C8B	1.491(4)
V1A-N1A	2.250(2)	C8B-C9B	1.382(4)
V1A-N2A	2.090(3)	C9B-C10B	1.373(5)

V1A-N3A	2.109(3)	C10B-C11B	1.386(5)
V1A-N4A	2.268(3)	C11B-C12B	1.366(5)
N1A-C1A	1.487(4)	C13B-C14B	1.490(4)
N1A-C7A	1.482(4)	C14B-C15B	1.390(4)
N1A-C13A	1.489(4)	C15B-C16B	1.378(5)
N2A-C2A	1.354(4)	C16B-C17B	1.380(5)
N2A-C6A	1.340(5)	C17B-C18B	1.376(5)
N3A-C8A	1.359(4)		

Symmetry codes: (i) 1-x, -y, 1-z

Table S2. Bond valence calculations for oxygen atoms in $[H_2V_{10}O_{28}]^{4-}$ for 1

V-O Bond	V-O length (*R*)	*s* = (*R*/1.791)$^{-5.1}$	Σ*s*
V1-O1	2.2268	0.32932	1.98708
V2-O1(i)	2.1390	0.40431	
V2-O1	2.1147	0.42857	
V3-O1	2.2884	0.28653	
V4-O1	2.3271	0.26305	
V5-O1	2.3064	0.27531	
V1-O2	2.0670	0.48145	1.86528
V2-O2	1.9162	0.70849	
V3-O2(i)	1.9343	0.67533	
V1-O3	1.7576	1.10077	1.75978
V4-O3	1.9436	0.65901	
V3-O4	1.7987	0.97836	1.79024
V4-O4	1.8657	0.81188	
V2-O5	1.6873	1.35552	1.81424
V4-O5(i)	2.0867	0.45871	
V1-O6	1.6111	1.71578	1.71578
V4-O7	1.6099	1.72232	1.72232
V3-O8	1.6063	1.74209	1.74209
V2-O9	1.6876	1.35429	1.86939
V5-O9	2.0398	0.51509	
V1-O10	1.9463	0.65436	1.22421
V5-O10	1.9998	0.56984	
V4-O11	1.7798	1.03251	1.84617
V5-O11	1.8649	0.81366	
V3-O12	1.9045	0.73098	1.78809
V5-O12	1.7716	1.05712	
V5-O13	1.6033	1.75878	1.75878
V1-O14	1.9203	0.70082	1.89813
V2-O14(i)	1.9465	0.65402	
V3-O14(i)	2.0186	0.54329	

Symmetry codes: (i) 1-x, -y, 1-z

1 checkCIF/PLATON report

1.1.1 Structure factors have been supplied for datablock(s) decatpa

THIS REPORT IS FOR GUIDANCE ONLY. IF USED AS PART OF A REVIEW PROCEDURE FOR PUBLICATION, IT SHOULD NOT REPLACE THE EXPERTISE OF AN EXPERIENCED CRYSTALLOGRAPHIC REFEREE.

No syntax errors were found. CIF dictionary Interpreting this report

Datablock: decatpa

Bond precision: C-C = 0.0053 A Wavelength=0.71073

Cell:	a=11.6736(10)	b=23.7172(17)	c=16.5914(12)
	alpha=90	beta=95.791(3)	gamma=90
Temperature:	300 K		

	Calculated	Reported
Volume	4570.1(6)	4570.1(6)
Space group	P 21/c	P 1 21/c 1
Hall group	-P 2ybc	-P 2ybc
Moiety formula	H2 O28 V10, 4(C18 H18 N4 O2 V), 4(H2 O)	H2 O28 V10, 4(C18 H18 N4O2 V), 4(H2 O)
Sum formula	C72 H82 N16 O40 V14	C72 H82 N16 O40 V14
Mr	2524.70	2524.69
Dx,g cm-3	1.835	1.835
Z	2	2
Mu (mm-1)	1.457	1.457
F000	2536.0	2536.0
F000’	2545.51	
h,k,lmax	15,32,22	15,32,22
Nref	11920	11854
Tmin,Tmax	0.840,0.864	0.660,0.746
Tmin’	0.840	

Correction method= # Reported T Limits: Tmin=0.660 Tmax=0.746 AbsCorr = NONE

Data completeness= 0.994 Theta(max)= 28.797

R(reflections)= 0.0393(8572) wR2(reflections)= 0.0917(11854)

S = 1.049 Npar= 640

```
The following ALERTS were generated. Each ALERT has the format
```
test-name_ALERT_alert-type_alert-level.

```
Click on the hyperlinks for more details of the test.
```

Alert level C

```
PLAT260_ALERT_2_C Large Average Ueq of Residue Including       O2W      0.170 Check
PLAT355_ALERT_3_C Long   O-H (X0.82,N0.98A)  O10       - H10      .      1.05 Ang.
PLAT910_ALERT_3_C Missing # of FCF Reflection(s) Below Theta(Min).          5 Note
PLAT911_ALERT_3_C Missing FCF Refl Between Thmin & STh/L=    0.600          6 Report
PLAT976_ALERT_2_C Check Calcd Resid. Dens.  0.57A   From O2W            -0.51 eA-3
PLAT976_ALERT_2_C Check Calcd Resid. Dens.  0.83A   From O2W            -0.50 eA-3
PLAT977_ALERT_2_C Check Negative Difference Density on H2WB             -0.50 eA-3
```

Alert level G

```
PLAT007_ALERT_5_G Number of Unrefined Donor-H Atoms ..............          5 Report
PLAT083_ALERT_2_G SHELXL Second Parameter in WGHT  Unusually Large       6.40 Why ?
PLAT720_ALERT_4_G Number of Unusual/Non-Standard Labels ..........         12 Note
PLAT764_ALERT_4_G Overcomplete CIF Bond List Detected (Rep/Expd) .       1.16 Ratio
 PLAT794_ALERT_5_G Tentative Bond Valency for V1        (V)       .       5.03 Info
 PLAT794_ALERT_5_G Tentative Bond Valency for V1A       (V)       .       5.36 Info
 PLAT794_ALERT_5_G Tentative Bond Valency for V1B       (V)       .       5.28 Info
 PLAT794_ALERT_5_G Tentative Bond Valency for V2        (V)       .       4.98 Info
 PLAT794_ALERT_5_G Tentative Bond Valency for V3        (V)       .       5.00 Info
 PLAT794_ALERT_5_G Tentative Bond Valency for V4        (V)       .       4.98 Info
 PLAT794_ALERT_5_G Tentative Bond Valency for V5        (V)       .       5.02 Info
PLAT883_ALERT_1_G  No  Info/Value  for  _atom_sites_solution_primary  .  Please  Do  !
PLAT912_ALERT_4_G  Missing  #  of  FCF  Reflections  Above  STh/L=  0.600  56  Note
PLAT933_ALERT_2_G  Number  of  OMIT  Records  in  Embedded  .res  File  ...   1  Note
PLAT978_ALERT_2_G Number C-C Bonds with Positive Residual Density.          1 Info
```

---

0 **ALERT level A** = Most likely a serious problem - resolve or explain

0 **ALERT level B** = A potentially serious problem, consider carefully

```
 7 ALERT level C = Check. Ensure it is not caused by an omission or oversight
15 ALERT level G = General information/check it is not something unexpected

 1 ALERT type 1 CIF construction/syntax error, inconsistent or missing data
 7 ALERT type 2 Indicator that the structure model may be wrong or deficient
 3 ALERT type 3 Indicator that the structure quality may be low
 3 ALERT type 4 Improvement, methodology, query or suggestion
 8 ALERT type 5 Informative message, check
```

Validation response form

1.1.2 Please find below a validation response form (VRF) that can be filled in and pasted into your CIF.

```
# start Validation Reply Form
_vrf_PLAT260_decatpa
```

```
;
PROBLEM: Large Average Ueq of Residue Including       O2W      0.170 Check
RESPONSE: ...
;
```

```
_vrf_PLAT355_decatpa
;
PROBLEM: Long   O-H (X0.82,N0.98A)  O10      - H10      .       1.05 Ang.
RESPONSE: ...
;
_vrf_PLAT910_decatpa
;
PROBLEM: Missing # of FCF Reflection(s) Below Theta(Min).          5 Note
RESPONSE: ...
;
_vrf_PLAT911_decatpa
;
PROBLEM: Missing FCF Refl Between Thmin & STh/L=    0.600          6 Report
RESPONSE: ...
;
_vrf_PLAT976_decatpa
;
PROBLEM: Check Calcd Resid. Dens.  0.57A   From O2W            -0.51 eA-3
RESPONSE: ...
;
_vrf_PLAT977_decatpa
;
PROBLEM: Check Negative Difference Density on H2WB             -0.50 eA-3
RESPONSE: ...
;
# end Validation Reply Form
```

1.1.3 It is advisable to attempt to resolve as many as possible of the alerts in all categories. Often the minor alerts point to easily fixed oversights, errors and omissions in your CIF or refinement strategy, so attention to these fine details can be worthwhile. In order to resolve some of the more serious problems it may be necessary to carry out additional measurements or structure refinements. However, the purpose of your study may justify the reported deviations and the more serious of these should normally be commented upon in the discussion or experimental section of a paper or in the "special_details" fields of the CIF. checkCIF was carefully designed to identify outliers and unusual parameters, but every test has its limitations and alerts that are not important in a particular case may appear. Conversely, the absence of alerts does not guarantee there are no aspects of the results needing attention. It is up to the individual to critically assess their own results and, if necessary, seek expert advice.

Publication of your CIF in IUCr journals

A basic structural check has been run on your CIF. These basic checks will be run on all CIFs submitted for publication in IUCr journals (*Acta Crystallographica*, *Journal of Applied Crystallography*, *Journal of Synchrotron Radiation*); however, if you intend to submit to *Acta Crystallographica Section C* or *E* or *IUCrData*, you should make sure that full publication checks are run on the final version of your CIF prior to submission.

1.2 Publication of your CIF in other journals

1.2.1 Please refer to the *Notes for Authors* of the relevant journal for any special instructions relating to CIF submission.

PLATON version of 13/07/2021; check.def file version of 13/07/2021

Datablock decatpa - ellipsoid plot

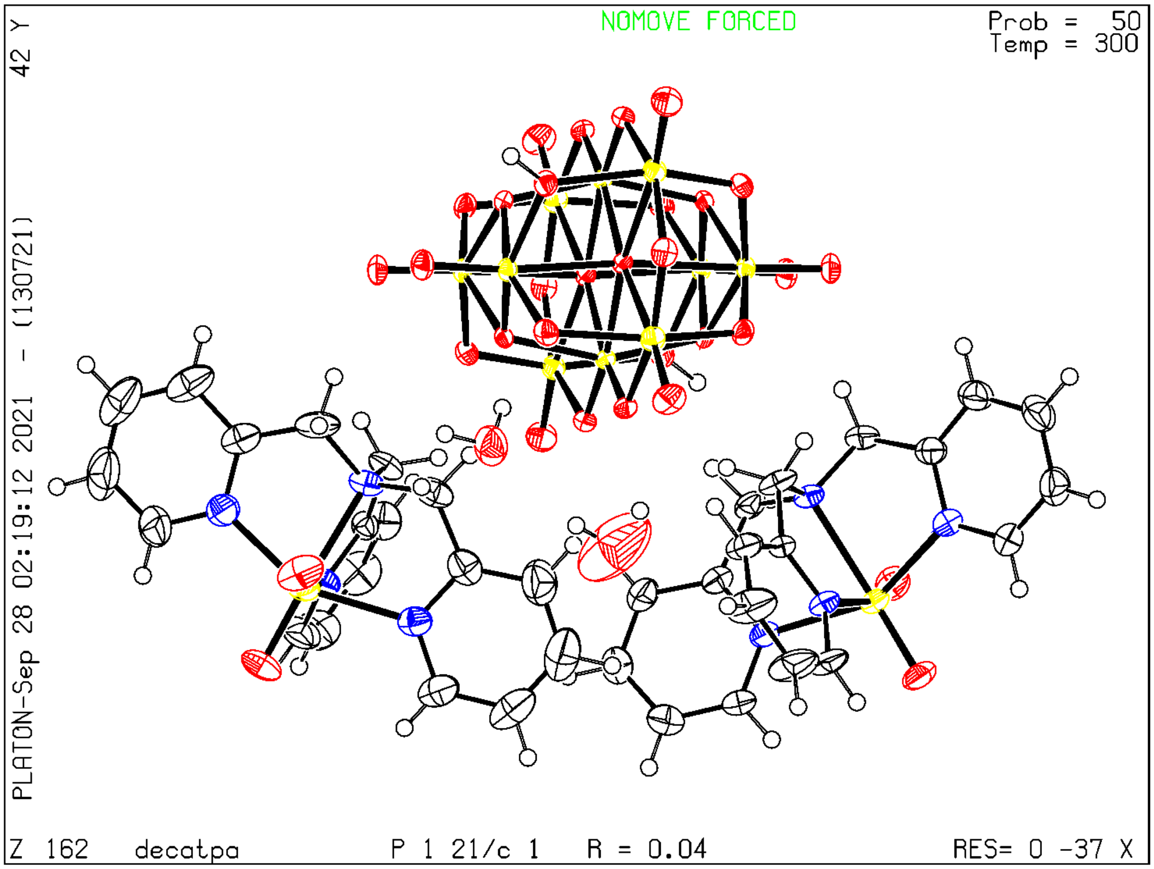

Supplement: Supplementary file 1 [file DataSheet1.pdf]
